# Globally-Optimal Inlier Maximization for Relative Pose Estimation Under Planar Motion

**DOI:** 10.3389/fnbot.2022.820703

**Published:** 2022-03-03

**Authors:** Haotian Liu, Guang Chen, Yinlong Liu, Zichen Liang, Ruiqi Zhang, Alois Knoll

**Affiliations:** ^1^State Key Laboratory of Vehicle NVH and Safety Technology, Chongqing, China; ^2^School of Automotive Studies, Tongji University, Shanghai, China; ^3^Robotics, Artificial Intelligence and Real-Time Systems, TUM Department of Informatics, Technical University of Munich, Munich, Germany

**Keywords:** Branch-and-Bound (BnB), Automated Guided Vehicle (AGV), relative pose estimation, inlier set maximization, rotation and translation estimation

## Abstract

Planar motion constraint occurs in visual odometry (VO) and SLAM for Automated Guided Vehicles (AGVs) or mobile robots in general. Conventionally, two-point solvers can be nested to RANdom SAmple Consensus to reject outliers in real data, but the performance descends when the ratio of outliers goes high. This study proposes a globally-optimal Branch-and-Bound (BnB) solver for relative pose estimation under general planar motion, which aims to figure out the globally-optimal solution even under a quite noisy environment. Through reasonable modification of the motion equation, we decouple the relative pose into relative rotation and translation so that a simplified bounding strategy can be applied. It enhances the efficiency of the BnB technique. Experimental results support the global optimality and demonstrate that the proposed method performs more robustly than existing approaches. In addition, the proposed algorithm outperforms state-of-art methods in global optimality under the varying level of outliers.

## 1. Introduction

Last decades witness the rapid development of frame to frame relative pose estimation in the field of computer vision, especially in visual odometry (VO), SLAM (Mur-Artal et al., 2015; Mur-Artal and Tardós, 2017), structure-from-motion (Schonberger and Frahm, 2016), 3D action understanding (Chen et al., 2014, 2015), trajectory online adaption (Luo et al., 2020, 2021) and gesture recognition (Qi and Aliverti, 2019; Qi et al., 2021). Relative pose estimation solvers recover correct relative 3D rotation and translation of the camera based on feature matching of consecutive image pairs to support the mentioned above applications, which promotes the mutual development of pose estimation, AGVs, and mobile robotic technology. Therefore, improving the accuracy and robustness of these solvers is of high interest to researchers. In this study, we focus on tackling the problem under planar motion constraint, e.g., the on-road vehicle is equipped with a forward looking camera. Such kinematic constraint is quite common and practical for Automated Guided Vehicles (AGVs) and robots designed for many real applications.

In visual geometry, all degree-of-freedom (DoF) relative pose problems between consecutive frames can be dealt with from 2D-2D point correspondences. Basically, eight points are sufficient to recover relative pose in 5-DoF (Hartley and Zisserman, 2003) with epipolar geometry. This is because epipolar geometry can construct a cross-relationship between the matched 2D points from different frames by introducing the 3 × 3 essential matrix which is derived from relative rotation and translation matrices. Nister's 5-point method (Nistér, 2004) improves the efficiency of computation of relative pose in a minimal way. Exploiting other constraints such as homography, the number of necessary points can be reduced (Ding et al., 2020). If we employ other sensors such as IMUs or stereo cameras to obtain auxiliary information, the minimal number of necessary points will descend to a lower level (Liu et al., 2016). Extremely, the Ackermann steering model constrains the car to move around a planar circle, therefore, one point correspondence is sufficient to recover the planar motion (Scaramuzza et al., 2009). The assumption that the camera moves around a planar circle limits the practical application of the Ackermann steering model. To solve this problem, we study the case that the camera moves under general planar motion. In our model, planar motion constraint simply descends the DoF of the problem to three, contributing to efficient modeling and computation.

Common solutions to the relative pose estimation problem are conducted based on accurate point correspondences (Nistér, 2004). However, real feature matchings are easily influenced by image noise and mismatches, which may lead to incorrect solutions. The common techniques to manage outliers rejection are RANdom SAmple Consensus (RANSAC) and its improvements (Fischler and Bolles, 1981; Raguram et al., 2013; Barath and Matas, 2018; Barath et al., 2019). Specifically, RANSAC is formulated to find consensus maximization (inlier maximization). The inlier is defined as a point correspondence satisfying the true relative pose in noisy input. That means the bigger the inlier subset is, the closer to the optimal solution the estimation will be. By setting a judging criterion, RANSAC tries to reserve the biggest subset of such point correspondences through iterations. Besides, many of its improvements (Raguram et al., 2013; Barath and Matas, 2018; Barath et al., 2019) are proposed to enhance the performance. Unfortunately, the number of iterations in RANSAC depends on levels of outliers while outliers are usually unknown. Therefore, in real applications, the number of iterations is usually fixed in advance by estimating the level of outliers, and if the parameter is over- or under-estimated, it may lead to redundant time-consuming or inadequate sampling iterations. More importantly, due to the heuristic nature, RANSAC and its improvements cannot provide a certifiably optimal solution for the object (i.e., inlier maximization) and may provide incorrect solutions or failures in some cases (Chin and Suter, 2017).

In this study, we propose a novel Branch-and-Bound (BnB) method to obtain globally-optimal inlier maximization for relative pose estimation under planar motion. To verify the feasibility and validity of the proposed method, we set several experiments on synthetic and real data. Different types of noise and varying levels of outliers are taken into consideration. Besides, performances on two real datasets KITTI (Geiger et al., 2012) and Malaga (Blanco-Claraco et al., 2014) show the strong robustness of the proposed approach. The main contributions are as follows:

- We propose a globally-optimal BnB algorithm for the relative pose problem under planar motion constraint, where the algorithm is suitable for mobile robots or AGVs.
- Owing to the special modification of motion equations, the relative pose can be decoupled into planar rotation and translation, enhancing the efficiency of the BnB technique greatly.
- Our experimental results show that the proposed method keeps better robustness under both image noise and outliers.

The rest of this study is organized as follows. Related study is reviewed in Section 2. Brief notations and the main algorithm are given in Section 3. In Section 4, comprehensive experiments on synthetic and real data are conducted to evaluate the performance of our BnB approach. Finally, we conclude our study in Section 5.

## 2. Related Work

Epipolar geometry is utilized to deal with the 5-DoF relative pose problem in multi-view geometry. It introduces the essential matrix to describe the relationship between different views and projected points. Basically, 8 points are sufficient to deal with the 5-DoF relative pose problem (Hartley and Zisserman, 2003). Considering the characteristics of the essential matrix, Nistér (2004) extends the study and proves that 5 points are enough to recover the essential matrix. Kneip et al. (2012) propose a novel epipolar constraint and estimates 3-DoF relative rotation independently of translation. Moreover, for globally-optimal inlier maximization, Yang et al. (2014) give a general BnB framework for essential matrix estimation, whose search space consists of a 5D direct product space of a solid 2D disk and a solid 3D ball. Similarly, Bazin et al. (2014) estimate 3-DoF relative rotation and focal length by the BnB technique without considering translation. Bazin et al. (2012) offer a BnB framework by rotation search, which performs well in 3D rotation estimation without considering translation. In general planar scenes, the DoF descends to 2 and many minimal solvers emerge (Chou and Wang, 2015; Hong et al., 2016; Choi and Kim, 2018). Choi and Kim (2018) propose two solvers to the equations of epipolar constraint by dealing with intersections of an ellipse and a circle or intersections of a line and a circle. Chou and Wang (2015) propose a method especially for the relative pose problem under large viewpoint changes under planar constraints. Besides, Scaramuzza et al. (2009) propose 1-DoF restrictive model by Ackermann steering model, which constrains the vehicle under nonholonomic movement so that instantaneous circular motion can be applied to the camera. While point-based methods combined with RANSAC provide a fast and feasible approach to relative pose, the global optimality cannot be fully guaranteed. Exploiting the BnB technique, our proposed method under similar planar constraints offers better robustness in outlier rejection. Similarly, using the restrictive Ackermann steering model, Gao et al. (2020) propose a globally-optimal solution under planar motion by the BnB technique which is the most relevant study to ours. In the study of Gao et al. (2020), the camera faces downward to fix depth so that homography can also be applied. Through parameterizing planar rotation and translation into trigonometric functions, the researcher computes the bound in each branch efficiently. In contrast, our study cancels this restrictive steering model and extends the scene of homography to general planar scenes, further improving the flexibility and practicality. Even in the general planar case, a refined trigonometric representation for bound computing is provided as well without any extra burden on computation.

Recently, some solvers (Raposo and Barreto, 2016; Barath and Hajder, 2018; Guan et al., 2020; Hajder and Barath, 2020) exploit affine correspondences to estimate relative pose. An affine correspondence consists of a pair of feature correspondence and a local affine transformation mapping the region of the first feature point to the surrounding region of the second one. The methods in Raposo and Barreto (2016), Barath and Hajder (2018) adopt affine correspondences rather than point correspondences to recover the essential matrix, which outperforms five points algorithm for 5-DoF relative pose estimation. Furthermore, Guan et al. (2020) exploit extra local affine transformation and joins it with epipolar constraint, leading to only one point correspondence needed for relative pose estimation under planar motion.

In addition to restricting the DoF of camera motions, the minimal feature matchings of the relative pose problem will descend as well when utilizing the additional sensors. Stereo sensors capture 2 images once and the disparity map can be computed to recover the depth information, which benefits to settling scale problem of relative translation. Besides, RGB-D sensors provide depth information directly. In terms of high DoF of camera motions, the methods in Xu et al. (2006) and Vakhitov et al. (2018) apply such sensors to improve the computation efficiency. Similarly, IMUs can be utilized to capture simultaneous angular velocity and acceleration. In actual applications, it offers accurate relative rotation with high frequency. Once the rotation is known, the general 5-DoF problem descends to 2. The method in Kneip et al. (2011) exploits 3D relative rotation information from inertial data in order to support further full pose estimation. Martyushev and Li (2020) propose minimal solvers to the problem of relative pose estimation with known relative rotation angle detected by a gyroscope. Fraundorfer et al. (2010) estimate relative pose with known 2 orientation angles. Sweeney et al. (2014) propose minimal solutions for determining the relative pose of generalized cameras given the axis of rotation which can be provided by IMU measurement.

## 3. Relative Pose Estimation Under Planar Motion Constraint

This section first illustrates epipolar geometry under planar motion constraint and then describes the proposed BnB method to search optimal parameters for the maximization of energy function in detail.

### 3.1. Epipolar Geometry Under Planar Motion Constraint

Epipolar geometry holds the ability to outline the inherent geometric relationship between two views, becoming the common tool to deal with relative pose problems. Algebraically, the 3 × 3 essential (or fundamental) matrix composed of relative rotation and translation is introduced to express the relationship with projected points. Given that a 3D point is projected on two normalized image planes, relative equations can be obtained exploiting epipolar geometry.


(1) $$\begin{array}{l} {\text{x}}_{2}^{T}\text{E}{\text{x}}_{1}=0 \end{array}$$

where ${\text{x}}_{2}={\left[u_{2},v_{2},1\right]}^{T}$ and ${\text{x}}_{1}={\left[u_{1},v_{1},1\right]}^{T}$ are normalized homogeneous coordinates of feature points in two views. **E** = [**t**]_×_**R**, in which **t** and **R** represent relative translation and rotation, respectively.

**Figure 1:**
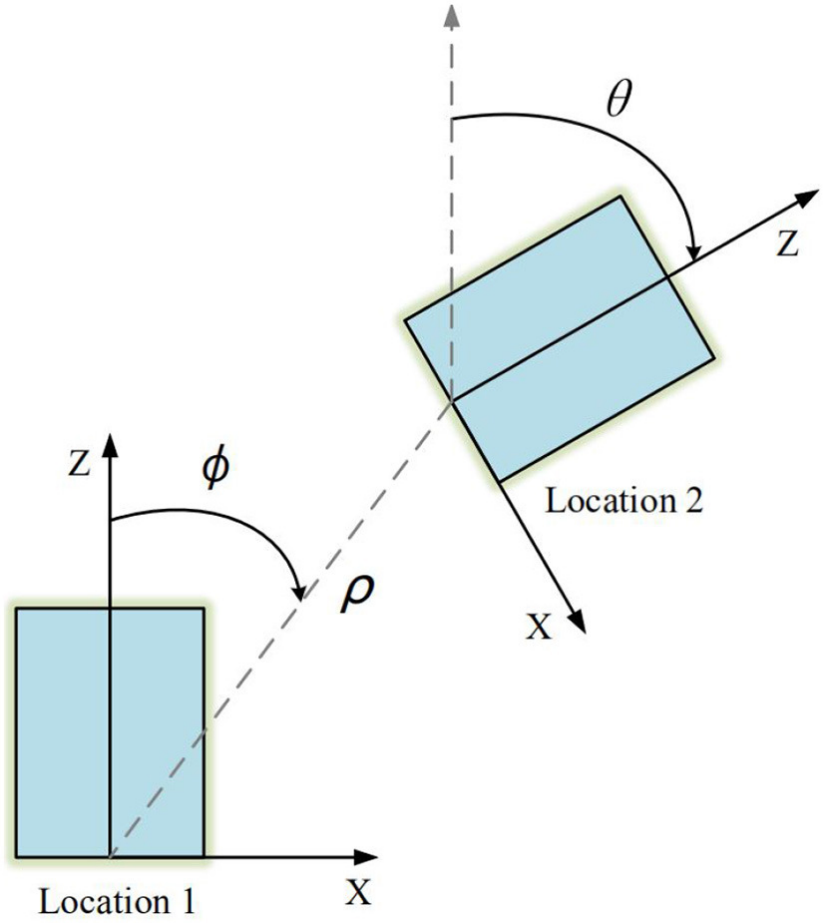
Planar motion from Location 1 to 2 in top-view. The relative pose can be described by θ and ϕ, where θ represents the yaw of the vehicle and ϕ represents the direction of translation. ρ denotes distance between two locations.

Intuitively, Figure 1 depicts a general scene under planar motion. We set the forward direction of the camera as *Z*-axis and the right direction as *X*-axis, so *Y*-axis points to the ground. Since the motion of the camera is constrained in planar scenes, the rotation matrix **R** = **R**_*y*_ under the view of Location 1 to 2 can be written as:


(2) $$\begin{array}{l} \text{R}={\text{R}}_{y}=\left[\begin{array}{ccc} \text{cos }\theta & 0 & -\text{sin }\theta \\ 0 & 1 & 0\\ \text{sin }\theta & 0 & \text{cos }\theta \end{array}\right], \end{array}$$

the translation matrix **t** can be written as:


(3) $$\begin{array}{l} \text{t}=-\text{R}\left[\begin{array}{c} \rho \text{ sin }\phi \\ 0\\ \rho \text{ cos }\phi \end{array}\right], \end{array}$$

then combining Equation 1 and **E** = [**t**]_×_**R**, we can gain the equation:


(4) $$\begin{array}{l} u_{1}v_{2}\text{ cos }\phi -v_{2}\text{ sin }\phi -u_{2}v_{1}\text{ cos }\left(\theta -\phi \right)-v_{1}\text{ sin }\left(\theta -\phi \right)=0. \end{array}$$

### 3.2. Proposed BnB Method

Let us observe the form of Equation 4. Drawing support from the auxiliary angle formula, we can rewrite the equation as:


(5) $$\begin{array}{l} A_{1}\text{ sin }\left(\theta_{1}+\phi_{1}\right)+A_{2}\text{ sin }\left(\theta_{2}+\phi_{2}\right)=0, \end{array}$$

where θ_1_ = θ − ϕ, ϕ_1_ = arctan(*u*_2_), θ_2_ = ϕ, ϕ_2_ = −arctan(*u*_1_), $A_{1}=v_{1}\sqrt{\left(1+u_{2}^{2}\right)}$, $A_{2}=v_{2}\sqrt{1+u_{1}^{2}}$. It is noted that such formulation of *A*_1_ and *A*_2_ is based on the assumption that *v*_1_ and *v*_2_ are non-negative. For negative *v*_1_ and *v*_2_, we just need to additionally discuss $A_{1}=-v_{1}\sqrt{\left(1+u_{2}^{2}\right)}$ and $A_{2}=-v_{2}\sqrt{1+u_{1}^{2}}$, of which the procedure is almost the same with the former. Without loss of generality, we simply assume that *v*_1_ and *v*_2_ are non-negative.

Next, given *M* feature correspondences from consecutive images in the normalized coordinate system, we build the energy function *g*(θ_1_, θ_2_) as:


(6) $$\begin{array}{l} g\left(\theta_{1},\theta_{2}\right)=\overset{M}{\underset{i=1}{\sum }}I\left(|A_{1}^{i}\text{sin}\left(\theta_{1}+\phi_{1}^{i}\right)+A_{2}^{i}\text{sin}\left(\theta_{2}+\phi_{2}^{i}\right)|<\varepsilon \right), \end{array}$$

where *I*(·) is an indicator function (which returns 1 if the condition is correct and 0, otherwise); ε denotes the tolerance considering unavoidable noise; superscript *i* denotes parameters from *i*th feature correspondence.

Our goal is to maximize function *g* by searching for the optimal θ_1_, θ_2_. However, the objective is non-smooth and non-concave, which means obtaining its optimal solution is not easy.

To obtain the optimal solution, we design a BnB algorithm, a globally-optimal solver based on search and iteration. By selecting branches of sub-problems with a higher priority which is estimated by well-designed bound strategies, BnB searches for globally optimal solutions efficiently. Algorithm 1 describes our BnB method to obtain globally-optimal relative pose under planar motion. Generally, we suppose θ_1_ ∈ *B*_1_, θ_2_ ∈ *B*_2_, and *B*_1_, *B*_2_ range from −π to π, respectively. For the branch step, we directly divide *B*_1_ and *B*_2_ into 2 equal parts uniformly. For the bound step, we first rewrite our objective function as:


(7) $$\begin{array}{l} f\left(\theta_{1},\theta_{2}\right)=\underset{\theta_{1},\theta_{2}}{\text{max}}g\left(\theta_{1},\theta_{2}\right), \end{array}$$

The lower bound and upper bounds are considered separately. It is evident that randomly selected θ_1_ and θ_2_ from *B*_1_ and *B*_2_ can comprise a lower bound *L*(*B*_1_, *B*_2_). Our objective function is to maximize *g*. For an upper bound *U*(*B*_1_, *B*_2_), given θ_1_ ∈ *B*_1_ and θ_2_ ∈ *B*_2_, we hope that


(8) $$\begin{array}{l} U\left(B_{1},B_{2}\right)\geq f\left(\theta_{1},\theta_{2}\right)=\underset{\theta_{1},\theta_{2}}{\text{max}}g\left(\theta_{1},\theta_{2}\right). \end{array}$$

To express more clearly, we denote


(9) $$\begin{array}{l} \rho_{i}\left(\theta_{1},\theta_{2}\right)=A_{1}^{i}\text{sin}\left(\theta_{1}+\phi_{2}^{i}\right)+A_{2}^{i}\text{sin}\left(\theta_{2}+\phi_{2}^{i}\right), \end{array}$$

that equals $U\left(B_{1},B_{2}\right)\geq \max\sum_{i=1}^{M}I\left(|\rho_{i}\left(\theta_{1},\theta_{2}\right)|<\varepsilon \right)$. The minimum and maximum of ρ_*i*_ can be expressed as:


(10) $$\begin{array}{l} \rho_{i}^{l}\leq \rho_{i}\left(\theta_{1},\theta_{2}\right)\leq \rho_{i}^{u}, \end{array}$$

then it is not hard to relax the indicator function


(11) $$\begin{array}{l} I\left(|\rho_{i}\left(\theta_{1},\theta_{2}\right)|<\varepsilon \right)=1 \end{array}$$

(12) $$\begin{array}{l} \Leftrightarrow I\left(-\varepsilon <\rho_{i}\left(\theta_{1},\theta_{2}\right)<\varepsilon \right)=1 \end{array}$$

(13) $$\begin{array}{l} \Rightarrow I\left(\rho_{i}^{l}<\varepsilon \text{ and }-\varepsilon <\rho_{i}^{u}\right)=1. \end{array}$$

Thus, the upper bound can be obtained as:


(14) $$\begin{array}{l} U\left(B_{1},B_{2}\right)=\overset{M}{\underset{i=1}{\sum }}I\left(\rho_{i}^{l}<\varepsilon \text{ and }-\varepsilon <\rho_{i}^{u}\right) \end{array}$$

(15) $$\begin{array}{l} \geq \underset{\theta_{1},\theta_{2}}{\text{max}}\overset{M}{\underset{i=1}{\sum }}I\left(|\rho_{i}\left(\theta_{1},\theta_{2}\right)|<\varepsilon \right) \end{array}$$

(16) $$\begin{array}{l} =f\left(\theta_{1},\theta_{2}\right). \end{array}$$

Note that the right side of the Equation 14 has no relation with θ_1_ and θ_2_, so the max operator can be aborted. Therefore, the remaining is to compute $\rho_{i}^{l}$ and $\rho_{i}^{u}$. Similarly, given θ_1_ ∈ *B*_1_ and θ_2_ ∈ *B*_2_, we just need to compute two minimum and maximum trigonometric functions separately.


(17) $$\begin{array}{l} \rho_{i}^{u}=\text{max }\rho_{i}=\text{max }A_{1}^{i}\text{sin }\left(\theta_{1}+\phi_{1}^{i}\right)+\text{max }A_{2}^{i}\text{sin }\left(\theta_{2}+\phi_{2}^{i}\right)\\ \rho_{i}^{l}=\text{min }\rho_{i}=\text{min }A_{1}^{i}\text{sin }\left(\theta_{1}+\phi_{1}^{i}\right)+\text{min }A_{2}^{i}\text{sin }\left(\theta_{2}+\phi_{2}^{i}\right). \end{array}$$

According to different range of *B*_1_, *B*_2_, and $\phi_{1}^{i}$ and $\phi_{2}^{i}$, the minimum and maximum can be achieved by category discussion. Gao et al. (2020) also parameterize relative rotation and translation by trigonometric functions, but we have different derivations, and please refer to Gao et al. (2020) for details.

It is worth noting that when *B*_1_ and *B*_2_ collapse to a single point, respectively, the upper bound and lower bound tend to be the same, ensuring the convergence of the proposed BnB method.

**Algorithm 1:**
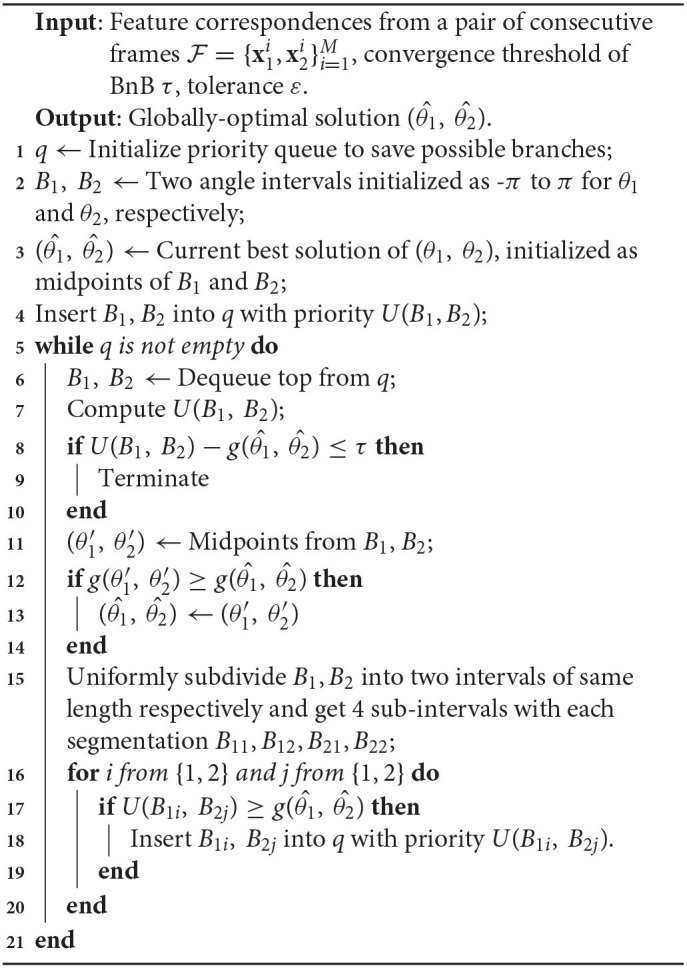
BnB for relative pose estimation from consecutive frames.

## 4. Experiments

In this section, we conduct experiments on synthetic and real data to evaluate the effectiveness and robustness of the proposed BnB method. To reject outliers, algorithms under comparison are combined with RANSAC. The parameters of RANSAC keep constant in the same experiment. All our experiments are executed on the Intel Core i7-9750H CPU. Our proposed BnB method is compiled and executed with C++ on Windows, The compared methods are written on Matlab R2020a, which may hold a slight difference from the original articles. Noting the randomness that existed in RANSAC, the estimated poses will not be fully consistent but quite close.

### 4.1. Experiments on Synthetic Data

We evaluate the effectiveness and robustness of our BnB method with synthetic data, respectively. The variances of the experiments are image noise and non-planar noise. Additionally, to evaluate the robustness and global optimality, we take an experiment under different ratios of outliers into consideration. Four different algorithms [1AC (Hartley and Zisserman, 2003), 2pt (Nistér, 2004), 5pt (Choi and Kim, 2018), 8pt (Guan et al., 2020)] using affine or point (feature) correspondences are computed for comparison.

To generate 3D points in space, we create 50 different virtual planes randomly and sample points distributed in the range of −5–5 m (*X* and *Y*-axis) and 10–30 m (*Z*-axis) on each plane. About 50 points are sampled in total. It is mentioned that (Barath and Kukelova, 2019) are introduced to estimate homography with four spatially close points from the same plane, after which local affine correspondence can be calculated to meet the requirement of extra affine information in 1AC. To simulate different views, we create 2 virtual cameras for which the focal length is 700 and the resolution is 1,000 × 400. Considering the scale problem in translation, we fix the distance between cameras by 2 m. With the assumption that the ground truth of relative pose is given by (θ_*gt*_, ϕ_*gt*_), we randomly choose them from $\left[-\frac{1}{3}\pi ,\frac{1}{3}\pi \right]$ to simulate the motion of autonomous vehicles. The candidates are fixed as (5, 5) degrees for simplicity. Thus, the rotation error and translation error between estimated parameters and precise ones can be defined as:


(18) $$\begin{array}{l} \varepsilon_{\text{R}}=|\theta -\theta_{gt}|,\varepsilon_{\text{t}}=|\phi -\phi_{gt}|. \end{array}$$

We replace the epipolar constraint Equation 4 with an inequality


(19) $$\begin{array}{l} |u_{1}v_{2}\text{cos }\phi -v_{2}\text{sin }\phi -u_{2}v_{1}\text{cos }\left(\theta -\phi \right)-v_{1}\text{sin }\left(\theta -\phi \right)|<\varepsilon , \end{array}$$

The inequality is exploited as a criterion for judging whether a pair of feature matching belongs to the set of the inliers. In all synthetic experiments, ε is fixed to 10^−3^. Besides, the number of iterations of the RANSAC scheme is decided by:


(20) $$\begin{array}{l} k=\frac{log\left(1-p\right)}{log\left(1-w^{n}\right)}, \end{array}$$

where *k* denotes the number of iterations, *p* the confidence, *w* the ratio of inliers, and *n* denotes the minimal cardinality of inlier set. In all synthetic experiments, we keep *p* as 0.9999 and except for the experiment under different outliers, *w* is taken as 0.8 since the image noise and non-planar noise are relatively small. Besides, all synthetic experiments are duplicated 200 times to reduce randomness.

**Figure 2:**
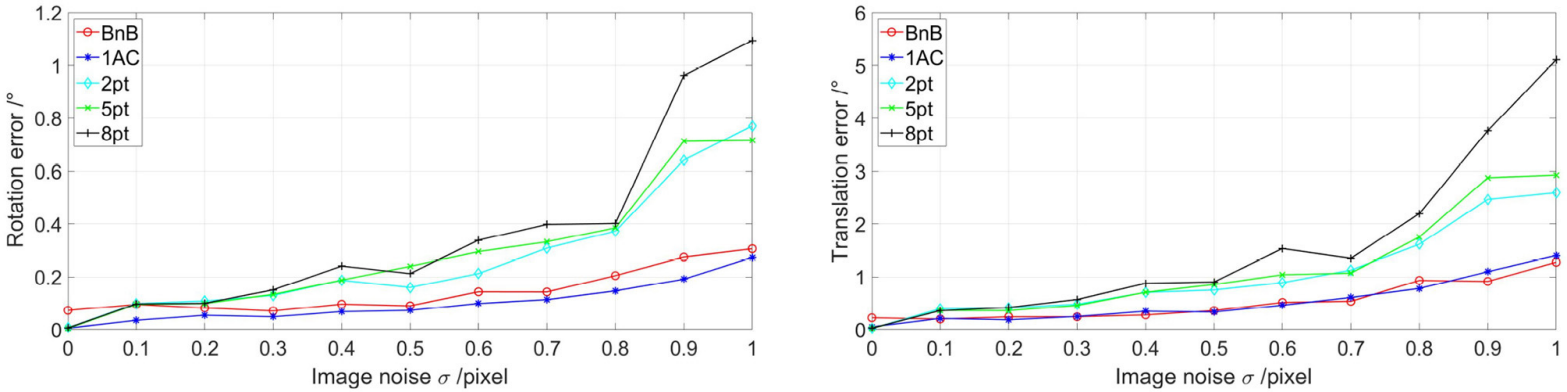
Evaluations of five algorithms on different image noise. The non-planar noise is not added. The left image shows rotation error with different image noise and the right one represents translation error with different image noise. 1AC, 2pt, 5pt, and 8pt are the studies of Hartley and Zisserman (2003); Nistér (2004); Choi and Kim (2018); Guan et al. (2020), respectively, and BnB is our study.

For experiments with the image noise as the variance, we set image noise with different Gaussian distributions *N*(0, σ^2^) with the SD σ ranging from 0 to 1. Under each σ, the median rotation and translation of 200 repetitions are utilized for evaluation. Figure 2 shows the performance under different image noises. Under small image noise, BnB, 1AC, and 8pt methods show competitive performances. Once the noise increases, the 8pt method falls behind while BnB and 1AC methods are stronger.

**Figure 3:**
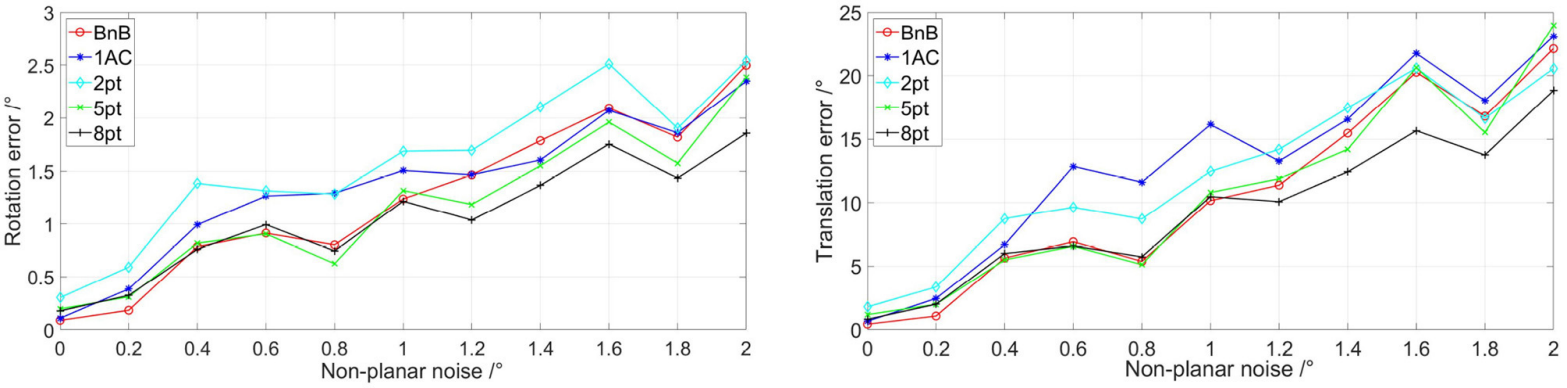
Evaluations of five algorithms on different non-planar noise. The image noise is set as σ = 0.5. The left image shows rotation error with different non-planar noise and the right one represents translation error with different non-planar noise. 1AC, 2pt, 5pt, and 8pt are the studies of Hartley and Zisserman (2003); Nistér (2004); Choi and Kim (2018); Guan et al. (2020), respectively, and BnB is our study. Better viewed in color.

Additionally, we add non-planar noise in rotation and translation to simulate more realistic road conditions. Following (Choi and Kim, 2018), the non-planar noise consists of *X*-axis rotation, *Z*-axis rotation, and the direction of *YZ*-plane translation. Similarly, the uniform noise is varied from 0 to 2 degrees. Besides, we fix the image noise with an SD of 0.5 pixels. Figure 3 shows the performances of the proposed BnB method with respect to non-planar noise. 8pt and 5pt methods perform well for the reason that they estimate 5-DoF relative pose. Besides, three other algorithms designed for planar cases show similar performance since they are poor to deal with non-planar noise well. Besides, the 1AC method shows similar performance on rotation compared with the proposed BnB method, while the BnB method outperforms it in translation estimation.

**Figure 4:**
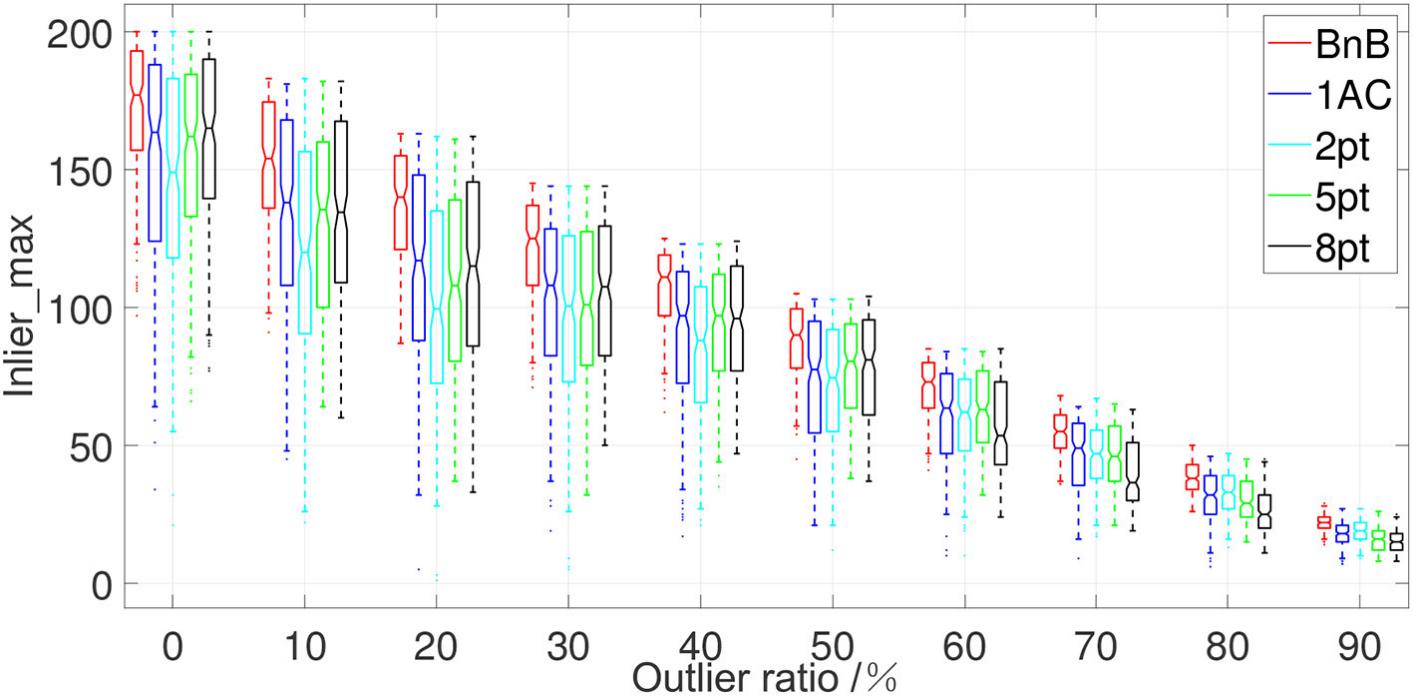
The boxplot of *inlier_max* of five algorithms with respect to different ratios of outliers. The horizontal axis represents the ratio of manually added outliers and the vertical axis represents the performance of the inlier maximization of five algorithms. 1AC, 2pt, 5pt, and 8pt are the studies of Hartley and Zisserman (2003); Nistér (2004); Choi and Kim (2018); Guan et al. (2020), respectively, and BnB is our study.

Apart from the image noise and non-planar noise, there exist many mismatches during feature matching, e.g., ASIFT and VLFeat. Since our BnB method aims to obtain a globally-optimal inlier maximization solution of the relative pose, we consider a common metric *inlier*_*max* (Chin and Suter, 2017) to evaluate the ability of inlier maximization. Specifically, *inlier*_*max* is defined as the maximal cardinality of the subset of inliers which satisfies Equation 19 and represents the global optimality of these methods. Given a noisy set of feature correspondences with mismatches, a globally-optimal solver should complete high-quality outliers rejection and keep a maximized subset of inliers. In detail, we fix the image noise with SD σ = 0.5 without considering non-planar noise. The sum of feature matchings is fixed to 200, and we add the different ratios of outliers in sampled feature matchings ranging from 0 to 90%. The median value of *inlier*_*max* in 200 repetitions is adopted for evaluation. Figure 4 shows the performance of the proposed BnB method under different ratios of outliers. It shows that when satisfying the criterion for judging whether a feature correspondence stands for correct relative motion, the proposed BnB method keeps the best inlier maximization under the different ratios of outliers compared with other solvers joined with RANSAC. As the ratio of outliers equals 70%, the proposed BnB method keeps the cardinality of inliers subset over 50 while other methods reserve inliers fewer than 50. This shows our proposed method manages to search for a globally-optimal solution even under heavy outliers and noise.

### 4.2. Experiments on Real Data

We evaluate the effectiveness and robustness of our BnB method mainly on the KITTI odometry dataset (Geiger et al., 2012) and Malaga dataset (Blanco-Claraco et al., 2014) for supplementation. KITTI odometry dataset contains 11 sequences with groundtruth of pose matrices from 00-10. We manage comprehensive evaluation through the full use of 11 sequences because the sequences cover different planar scenes. Since θ and ϕ are sufficient to describe the relative rotation and translation of the camera in consecutive frames without considering scale problems, we still compare ε_**R**_ and ε_**t**_. Besides, *inlier*_*max* is also used to evaluate the global optimality under mismatches in real datasets. For the Malaga dataset, we exploit *inlier*_*max* in that it does not provide groundtruth of camera poses.

**Figure 5:**
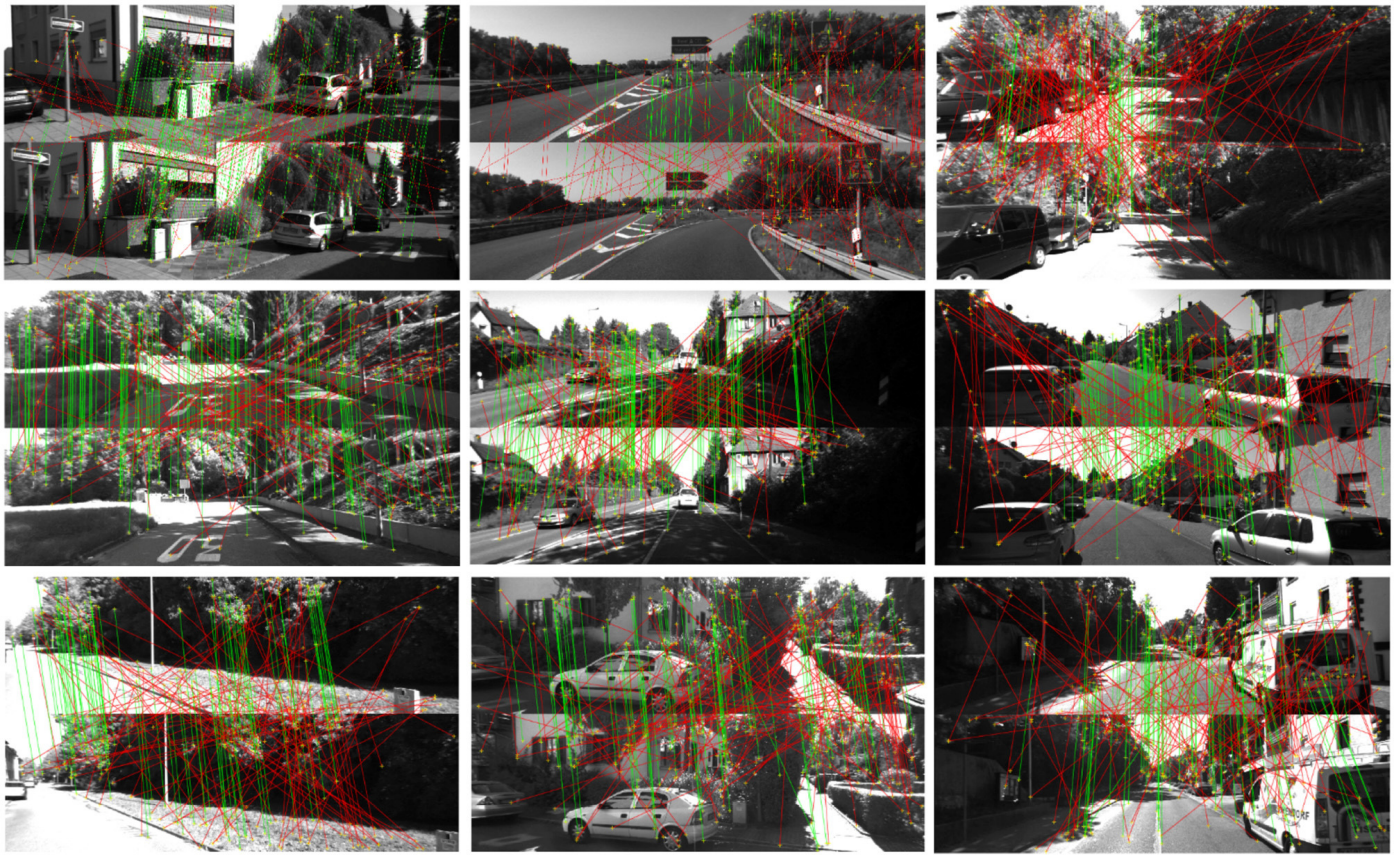
Visualization of night scenes using proposed BnB method on noisy matchings with sequences of KITTI odometry dataset. The green lines represent correct correspondences and the red lines denote mismatches. In each pair of scenes, the scene below moves from above. Better viewed in color.

The proposed BnB method is compared with 2 different algorithms [1AC (Choi and Kim, 2018), 2pt (Guan et al., 2020)] which are especially proposed for planar scenes. ASIFT (Morel and Yu, 2009) in VLFeat (Vedaldi and Fulkerson, 2010) is exploited to extract 50 affine correspondences between consecutive frames and the threshold of the matching scheme is set to 2 pixels. Besides, the tolerance ε of epipolar constraint is set to 10^−3^ since real data undergoes higher non-planar noise and mismatches. The number of iterations in RANSAC is fixed to 100 through experiments. For evaluating rotation and translation error, we take the median value on each sequence to avoid the influence of failures by RANSAC. The mean value of *inlier*_*max* is adopted to show the global optimality. Table 1 presents comparative results between the BnB method and 1AC (Choi and Kim, 2018), 2pt (Guan et al., 2020) methods on 11 KITTI odometry datasets. It shows that our BnB method provides a significant improvement in translation estimation in 10 of 11 KITTI sequences compared with 1AC and 2pt methods, where the smallest translation error is 0.0182 and the biggest one is 0.69. The biggest translation error of the 1AC and 2pt methods are 1.3076 and 4.2964, respectively, which are much worse than the proposed BnB method. Besides, the proposed BnB method obtains the highest *inlier*_*max* through all sequences and shows strong global optimality from the perspective of inlier maximization. Figure 5 exhibits some figures where the BnB method takes heavily noisy feature correspondences as input and achieves inlier maximization. The green lines denote inliers and red lines denote outliers caused by mismatches or big noise. As shown in Figure 5, green lines maintain a fairly consistent direction and the red lines intersect with each other, which meets the characteristics of proper correspondences and mismatches intuitively. In Figure 5, BnB reserves an inlier subset from extremely noisy matchings, and the green lines show high consistency, representing the relative motion between consecutive frames.

**Table 1:** Comparison of three methods on 11 sequences of KITTI odometry dataset.

**Seq**.	**ε**_**R**_			**ε**_**t**_			***inlier***_***max***		
	**BnB**	**1AC**	**2pt**	**BnB**	**1AC**	**2pt**	**BnB**	**1AC**	**2pt**
00	0.0337	**0.0139**	0.1956	**0.6900**	0.8346	3.7567	**41.5535**	39.3507	39.5037
01	0.0123	**0.0053**	0.1880	**0.0853**	0.2596	2.7532	**46.0509**	44.4045	45.0336
02	**0.0076**	0.0100	0.1510	**0.2973**	0.5691	2.2629	**43.2060**	40.9652	41.3803
03	0.0237	**0.0244**	0.1433	**0.6412**	1.3076	1.2426	**43.4563**	41.2638	41.5613
04	0.1231	**0.0272**	0.1270	**0.6663**	1.0309	1.7233	**46.8889**	45.0222	45.9269
05	0.0053	**0.0023**	0.1514	**0.0182**	0.1297	3.4481	**43.5569**	41.6315	41.7725
06	0.0427	**0.0148**	0.1611	**0.4229**	0.6721	2.5658	**44.3818**	42.2473	42.7400
07	**0.0011**	0.0033	0.1285	**0.0290**	0.3313	4.2962	**43.9855**	42.1764	42.3755
08	0.0100	**0.0018**	0.1374	0.0608	**0.0098**	3.0336	**43.5953**	41.5486	41.7359
09	0.0152	**0.0133**	0.1366	**0.0722**	0.5864	2.5314	**43.3050**	40.8648	41.4925
10	0.0076	**0.0051**	0.1391	**0.3161**	0.5296	3.0361	**43.0917**	40.8967	41.2442

*Seq represents the sequence number of the adopted data set. ε_R_, ε_t_, and inlier_max symbolize the rotation error, translation error, and average maximum matching point numbers. The bold values indicate the lowest error*.

**Figure 6:**
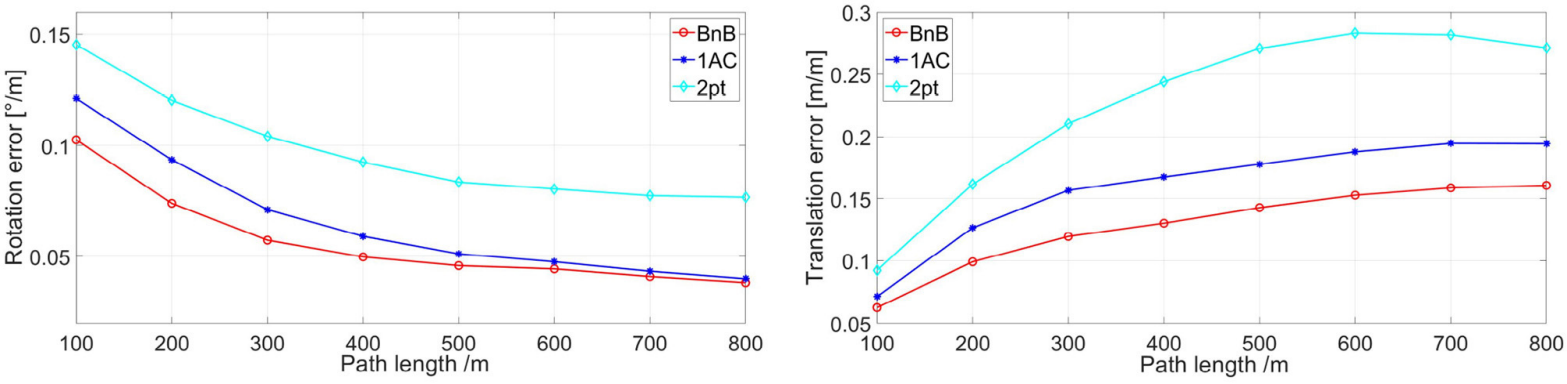
Rotation and translation error of three algorithms. They are exhibited with respect to path length. 1AC, 2pt are the studies of Choi and Kim (2018); Guan et al. (2020), respectively, and BnB is our study. Better viewed in color.

To give a comprehensive depiction of the performance of solvers above, we exhibit the relationship between the rotational and translation error defined in KITTI VO and SLAM Evaluation and the path length in Figure 6. For highlighting the capabilities of algorithms themselves, we estimate the distance ρ between consecutive frames from the groundtruth and do not manage any follow-up optimizations. The tolerance ε of epipolar constraint is decreased to 10^−4^ to show the performance more clearly. As shown in Figure 6, the BnB method shows higher performance in rotation error at the beginning and as the path gets longer up to 800 m, the rotation error tends to be consistent in BnB and 1AC methods. On the other hand, the translation error of the proposed BnB method is about 0.04 meter per meter less than the 1AC method, which shows an enhancement in translation error through the whole path length.

**Figure 7:**
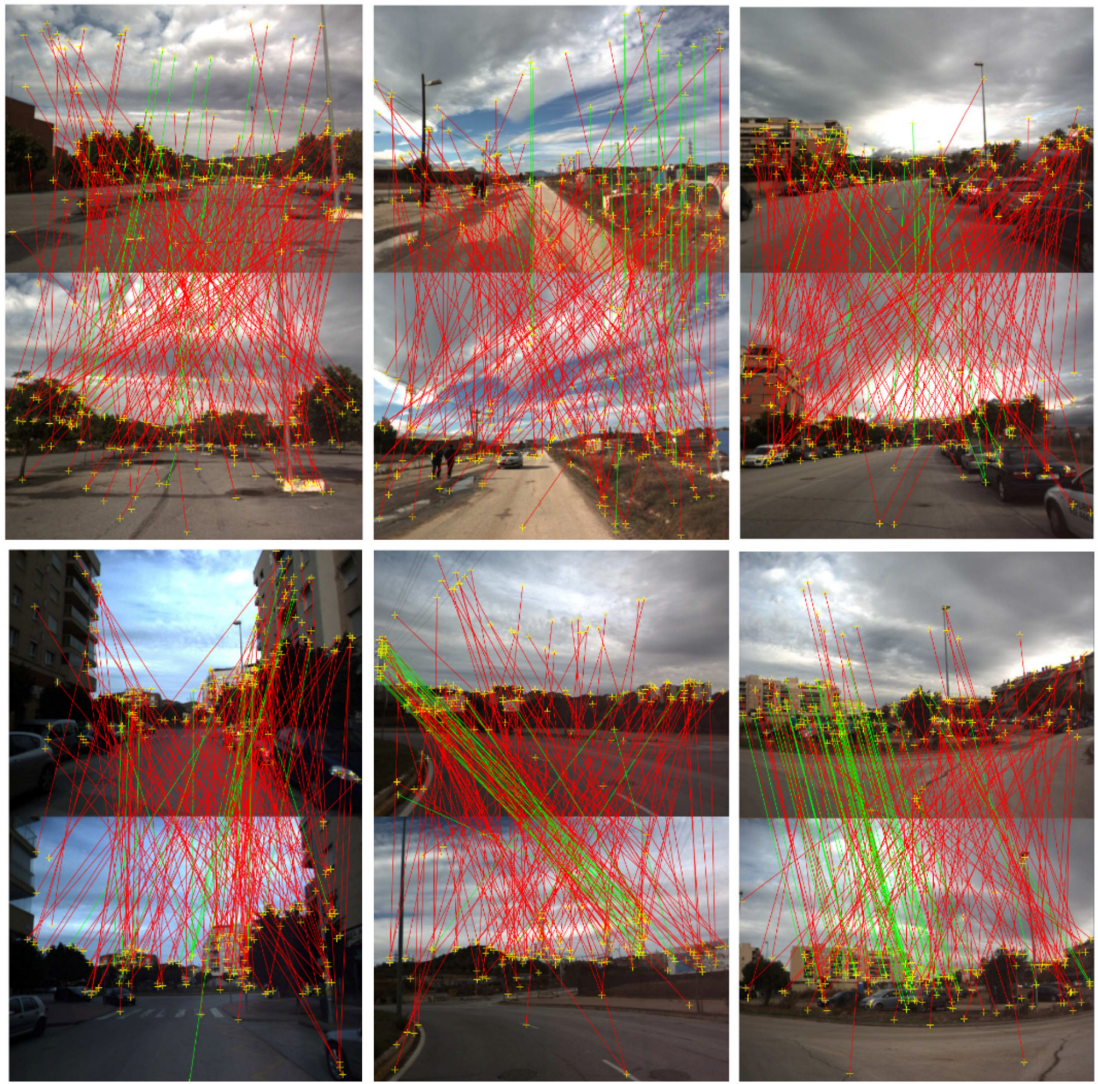
Inliers and outliers in five scenes of the Malaga dataset. The green lines represent correct correspondences and the red lines denote mismatches. In each pair of scenes, the scene below moves from above. Better viewed in color.

Besides, we randomly pick five scenes from the Malaga dataset in five different sequences to help evaluate the global optimality of the proposed method under noisy cases and *inlier*_*max* is exploited to evaluate the performance of three different methods. The tolerance ε of epipolar constraint is set to 10^−3^ and the number of RANSAC schemes is fixed to 1,000 to decrease the randomness. Table 2 shows the performance of three methods under different scenes. The feature correspondences are obtained from ASIFT and the matching threshold is 0.5 to add some mismatches. A total of 200 noisy feature matchings per example are randomly picked. As shown in Table 2, the five examples contain similar numbers of inliers, and the proposed method achieves the best inlier maximization in all scenes. It means that the proposed method keeps global optimality even under noisy cases. Figure 7 shows the five scenes of the Malaga dataset precessed by the proposed BnB method intuitively.

**Table 2:** Five pairs of consecutive frames selected from the Malaga dataset randomly.

**Scene *inlier_max* method**	**BnB**	**1AC**	**2pt**
Straight path	**22**	14	15
Through road	**23**	16	18
Roundabout	**18**	12	11
Roundabout with traffic	**21**	16	17
Loop closure	**27**	19	19

*We rename each scene, respectively, for simplicity. 1AC, and 2pt are the studies of Choi and Kim (2018); Guan et al. (2020), respectively, and BnB is our study. The bold values indicate the highest inlier maximization*.

In the end, due to the globally-optimal searching strategy of the proposed BnB method, the BnB method is more time-consuming than other non-minimal or minimal solvers. For 50 point correspondences from each pair of consecutive images and under the tolerance ε of epipolar constraint 10^−4^, BnB consumes 18.3203 s. While ε decreases to 10^−3^, consumed time decreases to 4.1867 s, and it also losses some precision.

## 5. Conclusion

Recent studies on relative pose estimation are targeted at more robust and faster methods, which will improve the performance of AGVs and robots. To enhance the robustness, we propose a novel globally-optimal BnB method for relative pose estimation of a camera under planar motion. Based on this reasonable assumption of planar motion for cameras fixed on self-driving cars or on-ground robots, our BnB method takes feature correspondences in the normalized camera coordinate system as input and obtains the globally-optimal solution for relative pose between consecutive frames effectively. Results of synthetic experiments show that our proposed BnB method has a highly effective performance of inlier maximization even on the high level of outliers. Additional experiments on the KITTI dataset and Malaga dataset both further confirm our BnB method is more robust than existing approaches. However, due to the globally-optimal searching strategy of the proposed BnB method, the proposed method is more time-consuming. For future study, we expect to find a tighter bound to speed up the convergence.

## Data Availability Statement

Publicly available datasets were analyzed in this study. This data can be found at: http://www.cvlibs.net/datasets/kitti/.

## Author Contributions

YL is responsible for ensuring that the descriptions are accurate and agreed by all authors and provided the original idea. The conceptualization and methodology were developed by ZL and HL. GC and AK: supervision and validation. RZ is responsible for software and visualization. All authors contributed to the article and approved the submitted version.

## Funding

This study was financially supported by State Key Laboratory of Vehicle NVH and Safety Technology 2020 Open Fund Grant, Project NVHSKL-202009, the GermanResearch Foundation (DFG), and the Technical University of Munich (TUM) in the framework of the Open Access Publishing Program.

## Conflict of Interest

The authors declare that the research was conducted in the absence of any commercial or financial relationships that could be construed as a potential conflict of interest.

## Publisher's Note

All claims expressed in this article are solely those of the authors and do not necessarily represent those of their affiliated organizations, or those of the publisher, the editors and the reviewers. Any product that may be evaluated in this article, or claim that may be made by its manufacturer, is not guaranteed or endorsed by the publisher.
